# A systematic review and meta-analysis of breastfeeding rates, factors influencing breastfeeding and practices in the United Arab Emirates (UAE)

**DOI:** 10.1186/s13006-025-00728-2

**Published:** 2025-05-16

**Authors:** Maeve Anne O’Connell, Shahla Meedya, Jenan Al Baqali, Kadeeja Alraeesi, Patricia Leahy-Warren

**Affiliations:** 1 Midwifery Department, Fatima College of Health Sciences, Abu Dhabi, United Arab Emirates; 2 Centre for Nursing and Midwifery Research, School of Nursing and Midwifery, Western Sydney University, Sydney, Australia; 3 Nursing Department, Awali Hospital, Awali, Bahrain; 4 Nursing Department, Brookfield School of Nursing & Midwifery, University College Cork, Cork, Ireland

**Keywords:** Breastfeeding, Prevalence, Trends, Infant, Infant feeding, Breastfeeding initiation, Exclusive breastfeed

## Abstract

**Background:**

Breastfeeding is recommended for optimal infant outcomes. Breastfeeding rates are thought to be suboptimal, but actual rates are not known in the United Arab Emirates (UAE). While there have been various studies about breastfeeding, there is no systematic review or meta-analysis in the UAE which has synthesized available evidence about breastfeeding rates and practices. The primary objectives of this study were to estimate breastfeeding rates, identify factors influencing breastfeeding, and explore breastfeeding practices among women in the United Arab Emirates (UAE).

**Methods:**

A systematic review and meta-analysis of relevant published peer-reviewed literature in six relevant electronic databases from 2013 to 1 August 2023. When statistical pooling was not possible, results were synthesized narratively.

**Results:**

Twelve studies were included. Pooled data from five studies (*n* = 2009) indicated that 62% of women initiated breastfeeding within the first hour after birth (95% CI 0.45, 0.78; I^2^ 98.3%). At three months, pooled data from three studies (*n* = 851) showed that 73% of these women were breastfeeding (95% CI 0.42, 0.96; I^2^ 98.7%). At six months, the rate of exclusive breastfeeding, based on pooled data from three studies (*n* = 1121), was 29.5% (95% CI 0.14, 0.477, I^2^ 97.5%). Significant heterogeneity was observed, suggesting that the results should be interpreted with caution. Three key themes emerged from the analysis of 12 studies: (1) balancing supportive factors with traditional practices, (2) the role of health service provision in breastfeeding, and (3) the influence of socioeconomic factors.

**Conclusion:**

This review highlights the importance of integrating cultural competence into healthcare strategies to better support breastfeeding mothers. Additionally, a national infant feeding survey is recommended to address the existing knowledge gaps in the UAE.

**Supplementary Information:**

The online version contains supplementary material available at 10.1186/s13006-025-00728-2.

## Background

Breastfeeding is widely recognized as the optimal method of infant feeding with short and long term benefits for mother and baby [1, 2]. The World Health Organization [3, 4] and United Nations Children’s Fund (UNICEF) recommend initiating breastfeeding within one hour of birth, and continued breastfeeding up to 2 years of age or beyond [3, 5]. EBF has been defined as offering only breast milk including expressed breast milk to infants up to six months of age; with exception of oral rehydration salts, nutritional supplements (vitamins & minerals) and medications if needed (WHO, 2008).

Globally, breastfeeding has been shown to improve both physical and mental health outcomes for mother and infant, regardless of the country’s income level [2, 6]. The United Nations’ Sustainable Development Goals (SDGs) emphasise the importance of breastfeeding as a preventative measure to save babies’ lives [7]. However, the aggressive marketing of formula which often violates the WHO International Code of Marketing of Breastmilk Substitutes poses a significant challenge to breastfeeding initiation and continuation [8]. The latest Lancet breastfeeding series calls for a collective commitment to improving breastfeeding practices at the population level rather than as simply the responsibility of women [8].

In the United Arab Emirates (UAE), rapid modernisation since its foundation in 1971 has led to shifts in traditional practices, including increased promotion of formula feeding and other infant food. The UAE government, however, has made efforts to prioritise breastfeeding in its national policies. The UAE’s National Strategy 2017–2021 aimed to increase breastfeeding rates from 34 to 50% for infants up to six months [9]. Recent media launches introduced the new UAE Nutrition Strategy (2022–2030), which continues to prioritize breastfeeding, although an English version of the strategy is not yet publicly available [10, 11]. UAE Federal Law mandates breastfeeding, with provisions for paid maternity leave of ninety days and an allowance of two continuous hours per day for breastfeeding upon return to work for six months [10, 11].

### Factors influencing breastfeeding

Women’s decision to initiate and continue breastfeeding are influenced by a variety of physical [12] and psychosocial factors [13]. A systematic review and meta-analysis of factors influencing breastfeeding in high income countries [12] reported smoking, mode of birth, parity, dyad separation, maternal education, and maternal breastfeeding education for determining initiation and duration of breastfeeding. Moreover, the context of a woman’s life, including her intentions, self-efficacy, and support systems, play a critical role in breastfeeding outcomes [13]. However, breastfeeding intention as the strongest predictor of breastfeeding initiation and continuation, can be influenced by women’s attitude, support and advice from family, friends and health professionals, sociocultural norms and social media [11].

In the UAE, breastfeeding is deeply intertwined with religious beliefs and cultural expectations among Muslim women, for whom motherhood and breastfeeding are seen as privileges and religious duties [11]. Breastfeeding also offers a natural family planning method through lactational amenorrhea [14]. Despite this, exclusive breastfeeding remains suboptimal in the UAE. Historically high breastfeeding rates have declined over the past thirty years. Recent studies show early initiation rates of 96% in Abu Dhabi [15], 72.5% in Dubai and Sharjah [16], and exclusive breastfeeding rates of 44% in Abu Dhabi (0–6 months) [15], 31% in Dubai (6 months to 2 years), and 22% in Sharjah (6 months to 2 years) [16]. Exclusive breastfeeding refers to infants receiving only breast milk, except for medications and vitamins, while early initiation is defined as infants latching within 1 h of birth [15, 16]. According to a review by Taha in 2017 [11], a National UAE 1988 infant feeding study reported that 88% of mothers breastfed, but mixed feeding has predominated since the 1970s.

A National Survey by the Ministry of Health and Prevention (MOHAP) conducted in 2017-18 reported that 92% of the 2361 women surveyed had ever breastfed [12]. However, the survey does not clearly define or calculate breastfeeding practices and notes that 61% of respondents gave birth in the UAE, leaving 39% who did not, which could skew the breastfeeding data specific to the UAE [12]. Therefore, the report provides only an estimation rather than an accurate assessment.

### Purpose of the review

This systematic review and meta-analysis aimed to estimate breastfeeding rates and synthesize evidence on factors influencing women’s breastfeeding practices in the UAE. By pooling data from various studies, the review provides evidence to inform policy and planning, potentially guiding interventions to improve breastfeeding rates.

## Methods

### Type of study and registration of protocol

This study was registered in the Prospective Register of Systematic Reviews (PROSPERO: CRD42023427736) and adheres to the Preferred Reporting Items for Systematic Reviews and Meta Analysis (PRISMA) statement (2021) [17] to support transparent reporting and aid the reader assess trustworthiness [18].

### Inclusion and exclusion criteria

This review was based on studies including adult women over 18 years old who ever breastfed residing in the UAE. We included all published peer-reviewed primary research articles in the English language, including observational studies, cross-sectional studies, cohort studies, prevalence studies, case control studies, randomized controlled trials and qualitative studies where data specific to breastfeeding could be extracted. Non-research letters, abstracts and editorials, seminar reviews and case studies/series reporting cases were excluded.

### Search strategy and selection procedures

**Fig. 1:**
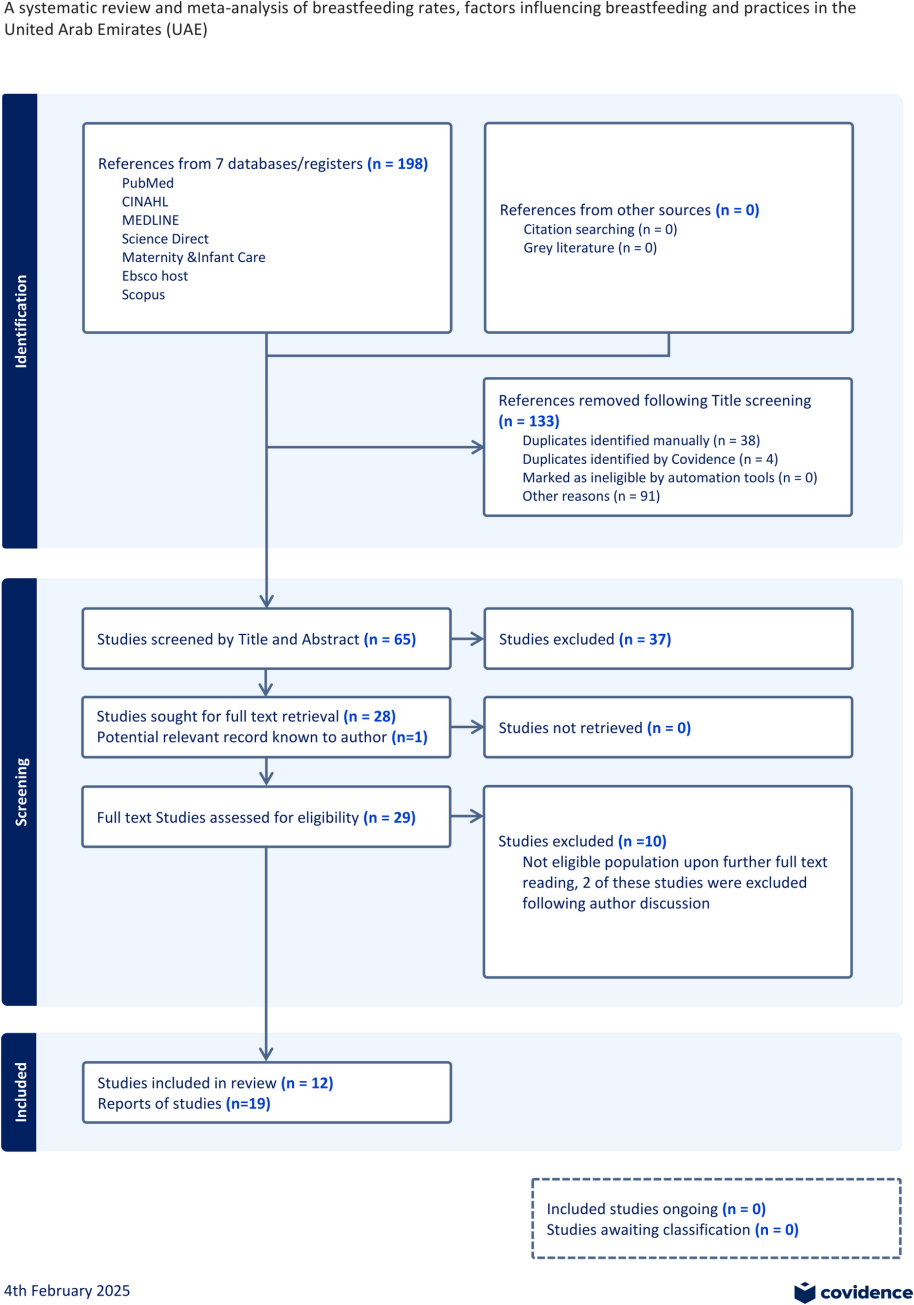
Prisma flow chart

Seven electronic databases were searched for relevant articles from 2013 to 1 August 2023 (Fig. 1). A search strategy developed with a health librarian (VC) was used. Records were imported into Clarivate EndNote version 20.5 library. Two researchers (MOC and SM) screened the studies and considered eligibility for the review. Reference lists of relevant articles were also hand searched for potential eligible studies.

The final list of included studies (*n* = 19) was then verified by two reviewers (MOC and SM). No disagreements arose during this process (Table 1).

**Table 1:** Study characteristics of included and excluded studies in the systematic review

Ref No	Study Author, Year	Design	Study Setting	Total *N*
	Included studies			6483
[20]	Ali et al., 2022	Retrospective survey	The Community. Recruitment via online survey promotion through social media to undisclosed recipients lists and by email to university students.	475
[21]	Al Ketbi et al., 2018	Cross-sectional	Four primary health centers in Abu Dhabi.	344
[16]	Al Sabbah et al., 2022	Cross-sectional	Outpatient clinics in four hospitals and four health centers in Sharjah and Dubai, UAE.	858
[28]	Al Shahwan et al., 2020	Cross-sectional	Breastfeeding mothers in Ajman and Sharjah, United Arab Emirates (UAE).	820
[22]	Cheikh Ismail et al., 2020	Population-based cross-sectional	Primary health care centers and outpatient clinics at hospitals.	276
[23]	Gardner et al., 2015	Cross-sectional	Tertiary Hospital: Postnatal Ward.	125
[24]	Gardner 2018	Cross-sectional	Tertiary Hospital: Postnatal Ward.	125*
[29]	Kaushal et al., 2022	Quality improvement prospective study	Hospital in Dubai.	
[25]	Omar et al., 2022	Cross-sectional	Four Primary Health Centers in Dubai.	668
[26]	Radwan 2013	Cross-sectional	Primary Health Care Centers in Abu Dhabi, Al Ain and Dubai.	593
[27]	Radwan et al. 2016	Qualitative	Maternal and Child Health, and Public Health Centers (PHC) in Abu Dhabi, Dubai and Al Ain.	45
[30]	Radwan et al. 2021	Prospective cohort	Maternity wards of ten hospitals in four Emirates (Abu Dhabi, Dubai, Sharjah, and Fujairah).	457
[15]	Taha et al. 2018	Cross-sectional	Seven governmental community and healthcare centers from diverse geographic areas of Abu Dhabi.	1822*
[34]	Taha et al. 2019	Cross-sectional	Seven governmental community and healthcare centers from diverse geographic areas of Abu Dhabi.	1624*
[32]	Taha et al. 2020	Cross-sectional	Seven governmental community and healthcare centers from diverse geographic areas of Abu Dhabi.	1822*
[36]	Taha et al. 2020 a	Cross-sectional	Seven governmental community and healthcare centers from diverse geographic areas of Abu Dhabi.	1822*
[33]	Taha et al. 2021	Cross-sectional	Seven governmental community and healthcare centers from diverse geographic areas of Abu Dhabi.	1822*
[31]	Taha et al. 2022	Cross-sectional	Seven governmental community and healthcare centers from diverse geographic areas of Abu Dhabi.	1822*
[35]	Taha et al. 2022 a	Cross-sectional	Seven governmental community and healthcare centers from diverse geographic areas of Abu Dhabi.	1799*
	**Excluded studies**		**Reason for exclusion**	
[55]	MOHAP 2018	National Survey	Not a peer reviewed publication and available data was estimated. Not clear which women in the survey gave birth in the UAE.	
[56]	Al Ghazal 2015	Health Promotion Report	Was not eligible upon reading full text: results of a health promotion campaign.	

*where multiple publications from the same study included, the largest subset was counted as the study population

### Data extraction

Two reviewers (MOC and SM) independently extracted the data using an evidence matrix (Table 2).

**Table 2:** Breastfeeding rates

Ref No	Study Author, Year	Emirate	Nationality	Total *N* (%)	Prenatal Intention to Breastfeed *n* (%)	Initiating Breastfeeding *n* (%)	EBF at 3 months *n* (%)	ABF at 3 months *n* (%)	EBF at 6 months *n* (%)	ABF at 6 months *n* (%)	EBF at 12 months *n* (%)	ABF at 12 months *n* (%)
[20]	Ali 2022	Northern Emirates (Ajman, Umm Al Quwain, Al Fujairah, Ras Al Khaimah	UAE Nationals & Non-Nationals	475 (100%)	Not reported	222 (47%)	Not reported	Not reported	222 (47%)	440 (93%)	Not reported	Not reported
[21]	Al Ketbi 2018	Abu Dhabi	UAE Nationals & Non-Nationals	354 (100%)	Not reported	193 (55%)	Not reported	Not reported	46 (13%)	Not reported	Not reported	Not reported
[16]	Al Sabbah et al., 2022	Dubai and Sharjah	Emirati or Arab	858 [492 in Dubai and 366 in Sharjah] (100%)	Not reported	214 (26%)*	Not reported	Not reported	Not reported	124 (14%)	Not reported as proportion	Not reported
[28]	Al Shahwan 2020	Sharjah and Ajman	UAE National & Non National	820 (100%)	Not reported	Not reported	Not reported	Not reported	Not reported	Not reported	Not reported	Not reported
[22]	Cheikh Ismail 2020	Dubai, Abu Dhabi and Sharjah	UAE National & Non- National	276 (100%)	Not reported	Not reported	135 (45%)	(94%)	11 (4%)	185 (67%)	1%	152 (55%)
[23]	Gardner 2015	Abu Dhabi	Emirati National	125 (100%)	116 (93%)	120 (96%)	5 (5%)	88 (70%)	0 (0%)	53 (42%)	0 (0%) **	26 (21%) **
[29]	Kaushal 2022	Dubai	UAE National & Non- National	n/a	Not reported	Not reported	Not reported	Not reported	Not reported	Not reported	Not reported	Not reported
[25]	Omar 2022	Dubai	UAE National & Non-National	668 (100%)	Not reported	503 (75%)	Not reported	Not reported	Not reported	Not reported	Not reported	Not reported
[26]	Radwan 2013	Dubai and Abu Dhabi/ Al Ain	UAE National	593 (100%)	Not reported	583 (98%)	Not reported	Not reported	Not reported	Not reported	Not reported	Not reported
[27]	Radwan 2016	Dubai, Abu Dhabi and Al Ain	UAE National	45 (100%)	Not reported	45 (100%)	Not reported	Not reported	Not reported	Not reported	Not reported	Not reported
[30]	Radwan 2021	Dubai, Abu Dhabi, Sharjah and Al Fujairah	UAE National & Non-National	457 (100%)	Not reported	316 (69%)	201 (50%)	372 (81%)	100 (22%)	201 (44%)	Not reported	Not reported
[15]	Taha 2018	Abu Dhabi	UAE National & Non-National	1822 (100%)	1655 (91%)	1741 (96%)	Not reported	154 (8%)	27 (1%)	Not reported	Not reported	8 (0%)

*EBF = Exclusive breastfeeding, ABF = Any breastfeeding

### Appraisal of methodological quality of studies

Methodological quality was assessed using the Mixed Method Appraisal Tool (MMAT) consisting of seven questions [27]. The reviewers (SM, MOC) independently classified the first two questions which are screening questions about having a clear research question. Then, they classified the other five questions which are more specific based on study design. if the first two screening questions were answered yes, the appraisal was processed for further evaluation (Additional File).

### Statistical analysis

**Fig. 2:**
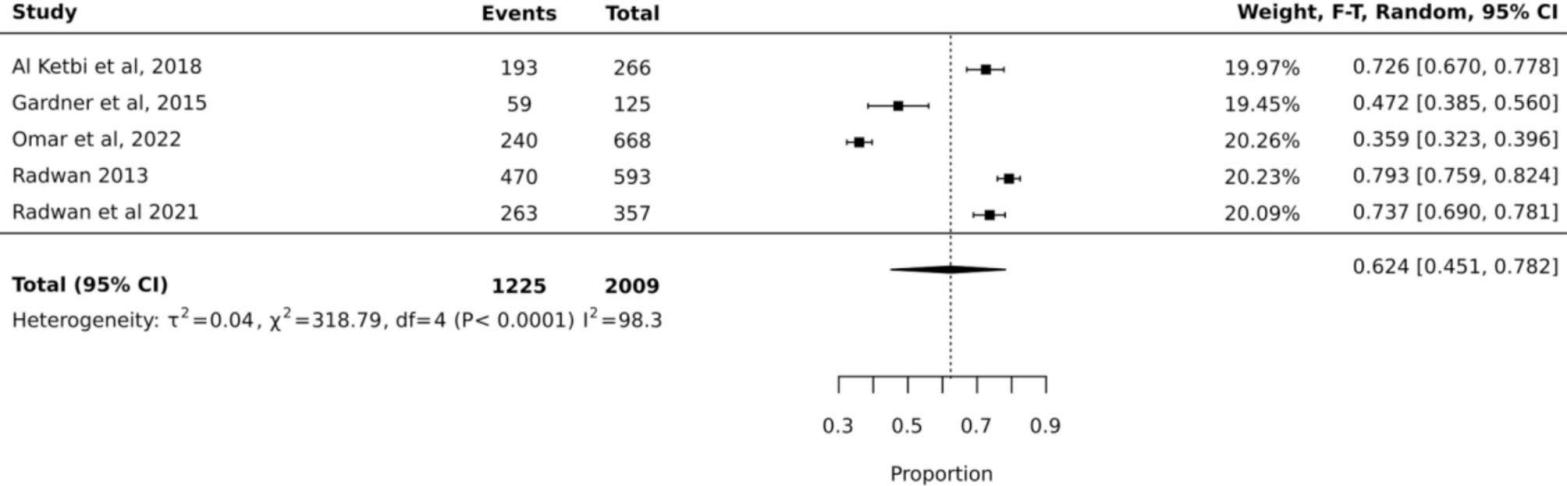
Forest plot for estimate prevalence of breastfeeding initiation at birth

For the prevalence, quantitative analysis was performed where data were available and pooled for a meta-analysis using a random effects model in Joanna Briggs Institute (JBI) System for the Unified Management of the Assessment and Review of Information (Summari) Software Package Version 15.0. The meta-analysis was conducted based on five categories: breastfeeding initiation, any breastfeeding at three, exclusive and any breastfeeding at six months. Effect sizes were expressed as odds ratios with calculated 95% confidence intervals. The presence of heterogeneity was tested using the standard I^2^ statistical test with a significance level of 90%. A forest plot was created to estimate prevalence of breastfeeding initiation at birth (Fig. 2), and findings of the meta-analysis presented in Table 3 (prevalence of breastfeeding initiation at birth, after one hour, at three months, six months and 12 months).

**Table 3:** Meta-analysis for breastfeeding outcomes

Outcomes	Number of Studies	Proportion of Women (%)	CI	I^2^
Breastfeeding outcomes				
Breastfeeding initiation in one hour	5 studies [21, 23, 25, 26, 30]	62.4	0.45, 0.78	98.3%
Breastfeeding initiation in more than one hour	5 studies [15, 23, 25, 26, 30]	24.6	0.18, 0.31	93.9%
Any breastfeeding at three months	3 studies [15, 23, 30]	72.9	0.42, 0.96	98.7%
Exclusive breastfeeding at six months	3 studies [20, 21, 30]	29.5	0.14, 0.477	97.5%
Any breastfeeding at six months	2 studies [20, 30]	85.9	0.74, 0.94	94.3%

For the related factors, narrative findings were synthesized through initially reading and re-reading the studies then using ‘Elicit software’ which is a novel machine-learning tool designed to assist in text extraction [19] and synthesis to tabulate concepts.

## Results

Results were compiled (*n* = 28), and duplicates (*n* = 1) removed (Fig. 1). Where there was more than one study on the same cohort of patients (same population), data were extracted from the study that described the whole population rather than a subset [15, 19–26].

### Characteristics of eligible studies

The search of electronic databases led to the retrieval of 198 study titles, irrelevant articles including duplicates were removed, resulting in 133 potentially eligible articles. The reading of titles and abstracts led the exclusion of 37 articles. Twenty-nine papers were submitted to full text analysis; 19 papers representing 12 studies met eligibility criteria and were included in the present systematic review (Fig. 1) and (Table 1).

Of the twelve studies, one was a retrospective survey [20], nine were cross-sectional [16, 21–28], one was a quality improvement study [29], and one was a longitudinal cohort study [30]. There was one qualitative study [27]. Results of two studies were included in multiple articles. A longitudinal study where women were interviewed at three, six and fifteen months postpartum published 2 articles [23, 24] and a cross-sectional multi-centre study which interviewed mothers of infants under the age of two years attending seven primary health centres in Abu Dhabi in 2017 published 7 articles [15, 31–36].

Overall, methodological quality was good based on the MMAT, with all studies meeting threshold criteria of 75% (Mean Score 98%) [37]. Total number of participants was 6483 and sample sizes ranged from 45 to 1822 (Table 2). All studies included women of mixed parity, both nulliparous and multiparous. Studies consisted of Emirati [15, 16, 20–23, 25–30] and Non-Emirati women [20–22, 25, 28–30].

### Factors that impact breastfeeding practices in the UAE

Following data analysis, three themes were identified: (1) balancing supportive factors with traditional practices; (2) health service provision for breastfeeding; and 3) socioeconomic influences.

### Themes

Themes were discussed and agreed between authors and presented as a narrative synthesis.

### Balancing supportive factors with traditional practices and modern recommendations

Supportive factors included strong perceived breastfeeding support from family and friends [29, 41], rooming-in to support night-time breastfeeding and not being offered formula in hospitals, education of women on breastfeeding benefits [34, 36, 39–41], with routine and timely follow-up [36, 38, 39]. Religious and cultural norms encourage women to breastfeed [34, 40, 42] and women perceive it to be clean and with minimal preparation [34]. However, support is often lacking, such as insufficient maternity leave or workplace breastfeeding facilities [21]. The necessity for many women to return to work after a short maternity leave of three months influences their breastfeeding plans. Two studies indicated that many mothers plan to use mixed feedings due to this need to return to work [15, 38].

Living with extended family can be challenging, especially when elders’ advice conflicts with breastfeeding practices [27]. Some grandmothers and mothers-in-law provide prelacteal feeds and additional foods like cereals, reinforcing the belief that mothers lack sufficient breastmilk [26]. Nevertheless, family members, particularly husbands, are key sources of information and support for breastfeeding surpassing health professionals [23, 27]. However, concerns regarding low milk supply and insufficient breastmilk lead to the introduction of early complementary feeding [33]. This is also compounded by the belief that infants could be thirsty in a hot climate and thus water is offered rather than breastmilk [27]. Furthermore, traditional cultural practices that do not support exclusive breastfeeding are challenging for women. For example, offering infants substances such as honey, herbal infusions, tea, yogurt, dates, and water during the first few hours and days of an infant’s life are practices rooted in cultural beliefs and customs [28]. Cross-nursing and wet-nursing, where infants are fed by women who are not their mother, add complexity to exclusive breastfeeding. While these practices provide breastmilk and are culturally acceptable, working mothers who lack this support may resort to mixed feeding with formula [14, 28], potentially leading to early cessation of breastfeeding.

### Health service provision for breastfeeding

Health policy support for exclusive breastfeeding was evident; the Ministry of Health issued an infant feeding policy which recommends six months of exclusive breastfeeding [26]. Furthermore, many hospitals have Baby Friendly Hospital Initiative (BFHI) accreditation, with an expectation of rooming in as part of the local policy in line with this [11, 29].

All those around the woman who give support- husband, family, friends, and maid - were influential to breastfeeding outcomes. The primary source of breastfeeding support was from family and a household maid [23, 27]. Lack of awareness about the importance of exclusive breastfeeding or recommended duration was common [27]. While support from husbands is helpful, men are usually not fully involved in infant feeding [27].

However, public perception is that health care providers are not as supportive as family [26, 29]. Barriers include healthcare staff’s insufficient knowledge, lack of rooming-in policies, and routine offering of formula, which undermine breastfeeding efforts [38, 40].

Common issues affecting the initiation of breastfeeding include insufficient skin to skin contact, delayed first breastfeeding session and lack of rooming-in [25, 29, 31, 34]. Additionally medical interventions such as Caesareans or separation from the baby at birth often contribute to delays in starting breastfeeding [25, 31, 34]. These practices impact the ability to establish a successful breastfeeding relationship early on [13, 39].

Some mothers reluctance to ‘room in’ posed difficulties in implementing baby-friendly policies in hospitals [29]. This reluctance often stemmed from cultural expectations that the baby would stay in a nursery and the common perception of staff and mothers of low milk supply [29].

### Socioeconomic influences

Four studies were encompassed in this theme [22, 29, 31, 34] covering issues such as older mother age, maternal obesity, Caesarean delivery, high income, education level, and employment. Breastfeeding challenges are exacerbated by maternal obesity.

### Meta-analysis results

Among studies with estimates of breastfeeding rates, the prevalence of breastfeeding initiation within the first hour of birth was 62% (95% CI 0.45, 0.78; I^2^ 98.3%) (*n* = 2009) [21, 23, 25, 26, 30] (Table 3). Pooled data from another 5 studies indicated an estimated prevalence of 25% (95% CI 0.18, 0.31; I^2^ = 93.9%) of women who initiated breastfeeding after one hour post birth [15, 23, 25, 26, 30](*n* = 3565). Exclusive breastfeeding rates at three months were reported in only two studies which were 5% [23], (*n* = 94) and 50% [30](*n* = 399). Pooled data from three studies [15, 23, 30] (*n* = 851) indicated an estimated rate of 73% of women with any breastfeeding at three months (95% CI 0.42, 0.96; I^2^ 98.7%). At six months, the rates of exclusive breastfeeding were pooled from three studies [20, 21, 30] (*n* = 1121) and the estimate was 29.5% (95% CI 0.14, 0.477; I^2^ 97.5%). The estimated rate of any breastfeeding at six months was 85.9% (95% CI 0.74, 0.94; I^2^ = 94.3%) [20, 30] (*n* = 849) [20, 21, 30]. One study reported any breastfeeding rate which was 50% at 12 months postpartum [23] (*n* = 52). Considering that definitions of breastfeeding vary across studies in our review, making comparisons is difficult (Table 3).

### Reasons for cessation

Overall, the most common reasons for stopping breastfeeding were perceived insufficient breastmilk production and inadequate maternity leave [21, 22, 28–30]. Other factors were lack of husband support [21], new pregnancy and that the infant weaned itself [23, 26, 28]. Poor infant weight gain and infant feeding issues were highlighted [20, 23, 25]. Sore or cracked nipples, pain and impaired infant sucking were also cited as reasons for cessation of breastfeeding in the studies [20, 25, 28, 31, 34]. Early cessation was found to be related to the need to return to work [28, 31] and having insufficient time during the workday for breastfeeding [21]. Other reasons for cessation were a personal decision, doctor or pharmacist advice, and artificial milk advertisements (media) [28].

More reasons for cessation were being advised not to breastfeed due to taking medications [25, 28], breastfeeding being too tiring or inconvenient or wanting to go on a weight loss diet [25]. Lack of lactational amenorrhea and using contraception were also linked to complementary feeding and early cessation [16].

## Discussion

This is the first systematic review and meta-analysis, to our knowledge, conducted to analyze breastfeeding practice in the UAE. Our review found a 62% rate of early breastfeeding initiation in the UAE which is similar in both developed and developing countries [21, 40]. Our review found a 29.5% rate of exclusive breastfeeding at six months in the UAE, which is considered fair by WHO definitions (as 0–11% as poor, 12–49% fair, 50–89% good and 90–100% very good) [41]. Our review synthesizes evidence on what factors influence women’s breastfeeding practices in the UAE. We compared our results with previous reviews, one review of the Middle East [42], Gulf Cooperation Council Countries (GCC) [43] and Saudi Arabia [40]. Our findings were similar to the findings of Alzaheb et al. who described factors associated with breastfeeding in the Middle East; mode of delivery, maternal employment, rooming-in, parity, pre-lacteal feeding, and other factors such as pregnancy planning, infants sex, breastfeeding substitute advertisements and night feeding [40, 42, 43].

The cultural tradition of introducing other fluids such as honey and water can interfere with breastfeeding [12, 43]. Including family and friends in prenatal education is essential as women often rely on their advice and support [35]. The GCC review recommended implementing BFHI policies and conducting country-specific studies to enhance breastfeeding knowledge in the region [43]. Ensuring that hospital staff do not introduce formula milk is important. Participatory Action Research (PAR) has been suggested as one method to drive change in clinical areas [44].

Understanding cultural dynamics is crucial for midwives to effectively support and educate mothers [45, 46]. Cultural competence involves tailoring interventions to fit the cultural contexts of patients served [45, 46]. In the UAE, religious expectations and legal mandates for breastfeeding for two years exist, yet many women are unaware of these supports [34]. Midwives should integrate cultural and legal knowledge, inform mothers about supportive factors, and dispel myths with evidence-based information that respects cultural beliefs. Involving husbands, mothers-in-law, and other key figures in breastfeeding discussions can create a more supportive environment and improve adherence to recommended practices [47]. Culturally competent midwives build trust and improve communication with patients [48]. However, the shortage of midwives in the UAE [49] affects infant feeding support, highlighting the need for midwifery education to include culturally appropriate care skills to ensure high-quality, respectful care in diverse settings [48].

This review identified returning to work as a major barrier to continued breastfeeding, underscoring the need for more research on the experiences of working women in the UAE. Non-supportive work environments are a common obstacle impeding breastfeeding efforts [7, 43, 50]. A lack of nurseries in most places of employment was identified as a barrier to continuing breastfeeding [16]. Without convenient access to childcare facilities, working mothers face difficulties in maintaining breastfeeding which may lead to early weaning. Implementing workplace policies which are supportive of breastfeeding such as designated lactation rooms and flexible breaks for breastfeeding mothers could mitigate these issues. Targeted research and interventions addressing the unique needs of working mothers in the UAE is needed.

The increasing preference for Caesarean was noted and the implications of this on early initiation of breastfeeding need to be considered [31, 34].

Support from healthcare professionals is crucial, yet there is a shortage of midwives who are key in providing breastfeeding support in various settings [34, 43]. The lack of follow-up support after hospital discharge further hinders sustained breastfeeding [28, 29].

These themes illustrate the complex factors contributing to delayed breastfeeding initiation and early cessation, having a negative impact on the health of mothers and their infants in the UAE, emphasizing the need for targeted interventions in hospital practices and policy implementation to support early breastfeeding.

A 2023 systematic review of Saudi women’s breastfeeding practices identified common reasons for stopping breastfeeding such as misconceptions around breastmilk sufficiency and returning to work [40]. In contrast, UAE studies suggest educating the whole family, not just childbearing women, is crucial. Unlike Saudi Arabia, where formula feeding and stopping for contraceptive use were noted, these were less prevalent in the UAE [40]. Theoretical approaches to interventions, incorporating familial and social factors, may enhance breastfeeding self-efficacy and intention [51, 52]. Future educational programs should incorporate the theories of breastfeeding self-efficacy and planned behaviour to promote sustained breastfeeding practices [52]. For instance, a Saudi Arabian study (*n* = 290), found that a tailored online intervention based on behaviour change models tripled exclusive breastfeeding rates at one month postpartum (66% vs. 26%, *p*-value < 0.001) [40].

### Recommendations

Education on key breastfeeding practices such as rooming in, avoiding formula and early initiation is crucial. The review highlights the need for breastfeeding support to extend beyond hospitals into the community, addressing the current lack of education and support after discharge [28, 29].

More research is needed on the experiences of women returning to work while breastfeeding in the UAE, as current evidence is limited. Although small studies exist, a National Infant Feeding survey is lacking. A National Infant Feeding survey would provide accurate data, particularly in the Northern Emirates and rural areas, enabling targeted intervention. The review by Al Nuaimi et al. recommended regular updates of key indicators of breastfeeding in each country of the GCC [43].

Developing education in relation to informed choice for Caesarean is a recommendation [34] since Caesarean birth may delay breastfeeding initiation and shorten exclusive breastfeeding duration and is considered the most significant factor influencing breastfeeding success [53].

### Limitations

The main limitation of this review is the type of study found. The studies were mainly cross-sectional conducted at one time point and used non standardized data collection methods. Our review revealed a lack of studies specifically exploring nulliparous women and their breastfeeding experiences and needs. All studies retrieved had a mixed parity study population. Nulliparous and parous women may have different needs in relation to breastfeeding practices [54]. This is an area which warrants further exploration in the UAE.

Studies conducted at various timepoints would yield more valuable information about breastfeeding prevalence. Most studies were conducted in the capital – Abu Dhabi. There may be cultural nuances observed in more rural areas of the UAE which would be beneficial to understand. Further research is required in the Northern Emirates and rural areas.

## Conclusion

This systematic review and meta-analysis, highlight good early breastfeeding rates in the UAE, but only fair exclusive breastfeeding rates at six months. To improve these rates, targeted interventions should address the various factors influencing breastfeeding. Key strategies include integrating cultural competence into midwifery education, involving family members in education and addressing workplace barriers. Focusing on these areas in future research and interventions is essential to support breastfeeding mothers and achieve WHO targets.

## Electronic supplementary material

Below is the link to the electronic supplementary material.

Additional file: Results of quality appraisal using the Mixed Methods Appraisal Tool (MMAT)

## Data Availability

No datasets were generated or analysed during the current study.
