# Supplementary material for: A systematic review and meta-analysis of breastfeeding rates, factors influencing breastfeeding and practices in the United Arab Emirates (UAE)
Source: Int Breastfeed J. 2025 May 16;20:37. doi: 10.1186/s13006-025-00728-2 (PMC12085000; doi:10.1186/s13006-025-00728-2)
Supplement: Supplementary file 1 — Additional file: Results of quality appraisal using the Mixed Methods Appraisal Tool (MMAT) [file 13006_2025_728_MOESM1_ESM.docx]

## Additional File. Results of Quality Appraisal using the Mixed Methods Appraisal Tool (MMAT)

| Author  Included Year | Criteria from the Mixed Methods Appraisal Tool (MMAT) | | | | | | | | | | | | | | | | | | | | | | | | | Overall Appraisal Value* |
| --- | --- | --- | --- | --- | --- | --- | --- | --- | --- | --- | --- | --- | --- | --- | --- | --- | --- | --- | --- | --- | --- | --- | --- | --- | --- | --- |
|  | 1.1 | 1.2 | 1.3 | 1.4 | 1.5 | 2.1 | 2.2 | 2.3 | 2.4 | 2.5 | 3.1 | 3.2 | 3.3 | 3.4 | 3.5 | 4.1 | 4.2 | 4.3 | 4.4 | 4.5 | 5.1 | 5.2 | 5.3 | 5.4 | 5.5 |  |
| Ali  2022 |  |  |  |  |  |  |  |  |  |  |  |  |  |  |  | 1 | 1 | 1 | 1 | 1 |  |  |  |  |  | 5 (100%) |
| Al Ketbi  2018 |  |  |  |  |  |  |  |  |  |  |  |  |  |  |  | 1 | 1 | 1 | 1 | 1 |  |  |  |  |  | 5 (100%) |
| Al Sabbah  2022 |  |  |  |  |  |  |  |  |  |  |  |  |  |  |  | 1 | 1 | 1 | 1 | 1 |  |  |  |  |  | 5 (100%) |
| Al-Shahwan  2020 |  |  |  |  |  |  |  |  |  |  |  |  |  |  |  | 1 | 1 | 1 | 1 | 1 |  |  |  |  |  | 5 (100%) |
| Cheikh Ismail  2022 |  |  |  |  |  |  |  |  |  |  |  |  |  |  |  | 1 | 1 | 1 | 1 | 1 |  |  |  |  |  | 5 (100%) |
| Gardner  2015 |  |  |  |  |  |  |  |  |  |  |  |  |  |  |  | 1 | 1 | 1 | 1 | 1 |  |  |  |  |  | 5 (100%) |
| Gardner  2018 |  |  |  |  |  |  |  |  |  |  |  |  |  |  |  | 1 | 1 | 1 | 1 | 1 |  |  |  |  |  | 5 (100%) |
| Kaushal  2022 |  |  |  |  |  |  |  |  |  |  | 1 | 1 | 1 | 0 | 1 |  |  |  |  |  |  |  |  |  |  | 4 (80%) |
| Omar  2022 |  |  |  |  |  |  |  |  |  |  |  |  |  |  |  | 1 | 1 | 1 | 1 | 1 |  |  |  |  |  | 5 (100%) |
| Radwan  2013 |  |  |  |  |  |  |  |  |  |  |  |  |  |  |  | 1 | 1 | 1 | 1 | 1 |  |  |  |  |  | 5 (100%) |
| Radwan 2021 |  |  |  |  |  |  |  |  |  |  |  |  |  |  |  | 1 | 1 | 1 | 1 | 1 |  |  |  |  |  | 5 (100%) |
| Radwan 2016 | 1 | 1 | 1 | 1 | 1 |  |  |  |  |  |  |  |  |  |  |  |  |  |  |  |  |  |  |  |  | 5 (100%) |
| Taha  2018, 2019, 2020,  2020a,  2021,  2022,  2022a |  |  |  |  |  |  |  |  |  |  |  |  |  |  |  | 1 | 1 | 1 | 1 | 1 |  |  |  |  |  | 5 (100%) |

*1 awarded for yes, 0 awarded for no
